# Mechanisms Involved in Dual Vasopressin/Apelin Neuron Dysfunction during Aging

**DOI:** 10.1371/journal.pone.0087421

**Published:** 2014-02-05

**Authors:** Julie Sauvant, Jean-Christophe Delpech, Karine Palin, Nadia De Mota, Jennifer Dudit, Agnès Aubert, Hélène Orcel, Pascale Roux, Sophie Layé, Françoise Moos, Catherine Llorens-Cortes, Agnès Nadjar

**Affiliations:** 1 Nutrition et Neurobiologie Intégrée, UMR 1286, INRA, Bordeaux, France; 2 Nutrition et Neurobiologie Intégrée, UMR 1286, Univ. Bordeaux, Bordeaux, France; 3 Center for Interdisciplinary Research in Biology (CIRB), U1050, INSERM, Collège de France, Université Pierre et Marie Curie-Paris VI, Paris, France; 4 Institut de GénomiqueFonctionnelle, PharmacologieMoléculaire, UMR 5203, CNRS, Montpellier, France; Univ. Kentucky, United States of America

## Abstract

Normal aging is associated with vasopressin neuron adaptation, but little is known about its effects on the release of apelin, an aquaretic peptide colocalized with vasopressin. We found that plasma vasopressin concentrations were higher and plasma apelin concentrations lower in aged rats than in younger adults. The response of AVP/apelin neurons to osmotic challenge was impaired in aged rats. The overactivity of vasopressin neurons was sustained partly by the increased expression of Transient receptor potential vanilloid2 (Trpv2), because central Trpv blocker injection reversed the age-induced increase in plasma vasopressin concentration without modifying plasma apelin concentration. The morphofunctional plasticity of the supraoptic nucleus neuron-astrocyte network normally observed during chronic dehydration in adults appeared to be impaired in aged rats as well. IL-6 overproduction by astrocytes and low-grade microglial neuroinflammation may contribute to the modification of neuronal functioning during aging. Indeed, central treatment with antibodies against IL-6 decreased plasma vasopressin levels and increased plasma apelin concentration toward the values observed in younger adults. Conversely, minocycline treatment (inhibiting microglial metabolism) did not affect plasma vasopressin concentration, but increased plasma apelin concentration toward control values for younger adults. This study is the first to demonstrate dual vasopressin/apelin adaptation mediated by inflammatory molecules and neuronal Trpv2, during aging.

## Introduction

Elderly individuals have a higher risk of becoming dehydrated than younger adults. Both lower levels of liquid intake and greater liquid losses contribute to dehydration in the elderly [1], [2]. Water reabsorption and excretion by the kidneys are regulated by circulating argininevasopressin (AVP) [3], [4] and apelin [5], [6] concentrations, respectively. These two neuropeptides are expressed by the same magnocellular neurons of the paraventricular (PVN) and supraoptic (SON) nuclei of the hypothalamo-neurohypophysial system [5], [7]. AVP is released from the posterior pituitary gland into the bloodstream in response to potent physiological stimuli, such as dehydration [3], [4], whereas apelin is released under hypoosmotic or water loading conditions [5], [8]. The opposite patterns of regulation of apelin and AVP have a biological purpose, maintaining water balance in the body by preventing additional water loss via the kidneys [9]. Interestingly, aging is accompanied by changes in AVP neuron activity, leading to an increase in the size of AVP-producing perikarya [10], [11], nucleoli [12] and Golgi apparatus [13], [14], high levels of *c-fos* messenger ribonucleic acid (mRNA) and high plasma AVP concentrations in aged animals [15]. This state of hyperactivation results in an increase in plasma AVP concentration [11]. However, no data are yet available concerning the release of apelin into the bloodstream during aging in euhydrated or dehydrated rats, and the molecular mechanisms responsible for changes in AVP/apelin neuron activity remain unknown.

Aging may also affect the intrinsic properties of neurons, modifying not only the basal activity of AVP/apelin neurons, but also their reactivity to physiological stimuli, such as dehydration. SON neuron excitability is subject to continual fine-tuning by calcium-permeable cation channels, the Trpvs. In particular, Trpv1, which is strongly expressed in many brain areas [16], has been shown to mediate osmosensing and thermosensing [17]. Within the SON, the osmotic and thermal control of magnocellular neurosecretory neurons is ensured by the N-terminal variant of Trpv1 [17]. Conversely, in adult rats, Trpv2 is expressed in a discrete manner, in very limited areas of brain nuclei, such as the PVN, suprachiasmatic nucleus and SON [18], all containing AVP neurons and displaying apelin immunoreactivity [6]. Furthermore, Trpv2 has been shown to be translocated upon cell activation, and neurons with Trpv2 immunoreactivity *in vivo* are known to engage in sporadic, intense activity [18]. Trpv2 is also modulated by inflammatory molecules, because systemic infection induces an increase in Trpv2 expression in the rat dorsal root ganglion [19]. AVP neurons are overactivated in aged rats. Trpv2 is thus a potential candidate molecule for involvement in neuronal adaptation of this type in an inflammatory context.

During chronic dehydration, the hypothalamo-neurohypophysial system, including the SON, undergoes a remarkable anatomical remodeling that is reversed by the cessation of stimulation [20], [21]. This remodeling is characterized principally by a pronounced decrease in the astrocyte coverage of magnocellular neurons and synapses, which regulates their activity [22], [23]. Consequently, water imbalance during aging probably results from both neuronal and glial cell dysfunction. Indeed, brain astrocytes and microglial cells and their immune functions are affected by aging but few data concern the aged SON.

However, it remains unknown whether the morphofunctional plasticity described in adult rats subjected to dehydration [20], [21] continues to operate in aged animals. Indeed, adult SON astrocytes are known to express both Glial Fibrillary Acidic Protein (GFAP), an intermediate filament providing cells with support and strength (marker of mature astrocytes) and vimentin, an intermediate filament protein (marker of immature astrocytes) conferring a certain degree of immaturity and morphological plasticity [24]. Following dehydration, astrocyte processes have been shown to retract and rearrange themselves in a manner similar to that in the ventral glia limitans (VGL) underlying the SON [25], [26], [27]. In turn, brain microglial cells are known to become overactive with age-related changes [28] and to increase in number [29], but no data concerning the aged SON are available yet. Aging has been also associated with altered immune functions [30], with lower levels of anti-inflammatory molecule production [31] and a more marked pro-inflammatory profile [32]. In aged SON, astrocyte hyperactivation [24] is accompanied with Interleukine-6 (IL-6) overproduction and a decrease in IGF-I concentration, this imbalance playing a role in AVP neuron activity [11]. On the other hand, the microglial cells, which are known to release many inflammatory molecules, including IL-1β and tumor necrosis factor alpha (TNF-α), in many brain areas [33] may also underlie the AVP/apelin neuronal adaptation in the aged SON especially as brain microglia has been shown to become activated with age-related changes [28].

In this work, using 3- and 22-month-old rats, we first analyzed and characterized the morphofunctional status of AVP/apelin neurons. We investigated their morphofunctional features, measuring the volume of the nucleus, and determining plasma AVP and apelin concentrations, under basal condition and after 48 h of dehydration, a chronic osmotic stimulus known to increase plasma AVP concentrations in younger adult rats [34], [35]. We further investigated whether neuronal Trpv2 expression was altered in the aged SON, and whether central treatment with a Trpv channel blocker restored the normal functioning of AVP/apelin neurons in aged rats as measured by plasma AVP and apelin concentrations. We used ruthenium red, a Trpv antagonist that blocks all Trpv subtypes, as no specific Trpv2 antagonist was available. We also examined the morphofunctional state of aged GFAP and vimentin-immunoreactive (IR) cells as well as their functional plasticity in response to 48 h dehydration, measuring in particular the width of the GFAP-IR and VIM-IR SON-VGL in both control and test conditions. We then tested whether IL-6 overproduction by aged astrocytes affected dual AVP/apelin neuron functioning by measuring the effects of a central injection of Il-6 antibody on plasma AVP and apelin concentrations and on Trpv2 neuronal expression. Microglial activation state was determined by carrying out a morphofunctional study based on staining for CD11b, a marker of activated microglial cells. The reactivity of microglial cells was estimated by inducing a transitory inflammatory state (lipopolysaccharide, LPS, treatment), and quantifying CD11b mRNA as well as IL-1β and TNF-α proteins levels in the SON of aged rats. We further investigated the involvement of aged microglia in neuron AVP/apelin adaptation, by evaluating the effects of treatment with minocycline, an inhibitor of microglial metabolism, on microglia astrocytes and AVP neurons.

## Methods

### Ethics statement

All animal experiments were conducted according to the INRA Quality Reference System, and to relevant French (Directive 87/148, Ministère de l'Agriculture et de la Pêche) and international (Directive 86/609, November 24th 1986, European Community) legislation. They adhered to protocols approved by Région Aquitaine Veterinary Services (Direction Départementale de la Protection des Animaux, approval ID: A33-063-920). Measures were taken to minimize pain, discomfort, and the number of animals used. Rats were handled daily for 1 week before the experiment onset to minimize stress reactions to manipulation.

### Animals

Male Wistar rats aged 3 (younger adults, referred to hereafter simply as “adult”) and 22 (aged) months were obtained from Janvier (France). They were initially housed in pairs, in transparent polycarbonate cages, in which they were allowed to acclimate to laboratory conditions for at least two weeks. They were maintained under standard colony conditions in a room with controlled temperature (23+/−1°C) and humidity (40%) and a 12-12 h light/dark cycle (light on at 7:00 am). Food (U.A.R., Epinay-sur-Orge, France) and water were provided *ad libitum*.

### Treatments

For studies of the functional reactivity of AVP neurons and astrocytes during aging, rats were given either *ad libitum* access to water (control) or were deprived of water for two days (48 h dehydration).

Surgical procedure for intracerebroventricular injections: Stereotaxic surgery was performed under anesthesia induced by an intraperitoneal (i.p.) injection of a mixture of ketamine and xylazine, at a dose of 0.1 ml per 100 g of body mass. Rats were placed in a Kopf stereotaxic instrument (Tujunga, CA, USA) and a stainless-steel guide cannula (23-gauge, 11 mm length) was implanted in the third ventricle (i.c.v; coordinates: AP: −0.8; L: 0; V: −7) [36]. Rats were allowed to recover for one week after implantation of the cannula, before the injection.

Drugs and treatments: Minocycline, an inhibitor of microglial metabolism, was used to study the possible role of microglia in the modulation of neuronal and astrocyte crosstalk in aged rats [11]. On the basis of published results [37] and pilot experiments, we administered minocycline hydrochloride (Sigma Aldrich, Saint-Louis, USA) diluted in phosphate-buffered saline (PBS) by i.p. injection, at a dose of 45mg/kg body weight, over five days. We investigated the potential role of IL-6 overproduction by astrocytes [15] in the alteration of neuronal and astrocyte crosstalk in aged rats, by assessing the effect of a central injection of IL-6 antibody (IL-6 Ab) on the morphofunctional status of AVP neurons and astrocytes, and on the functionality of AVP neurons, as attested by plasma AVP concentration. We induced inflammation with lipopolysaccharide (LPS, *Escherichia coli*, serotype 0127:B8, Sigma, Saint-Louis, USA) diluted in phosphate-buffered saline (PBS, 0.1 M). The pH of the buffer was adjusted to 7.4 and its osmolarity was 300±2 mOsm.L^−1^. It was prepared in endotoxin-free sterile water. For all experiments, LPS was injected i.p., at 9.00 a.m., at a concentration of 250 μg/kg body weight [38]. We neutralized IL-6 overproduction by astrocytes, by injecting a goat anti-rat IL-6 Ab (R & D Systems, Mineapolis, USA) i.c.v., as previously described [15]. The IL-6 Ab was dissolved in artificial cerebrospinal fluid (aCSF: 26.2 mM NaHCO_3_, 10 mM glucose, 120 mM NaCl, 1 mM Na_2_HPO_4_, 2.5 mM KCl, 1 mM MgCl_2_, 2.5 mM CaCl_2_). Given the constant of dissociation (*K*_d_) of IL-6 from its receptor (100 µg ml^−1^), we estimated that at least 2x*K*_d_ (200 ng/rat) concentrations of this neutralizing antibody would be required to observe an effect. The IL-6 Ab was administered i.c.v. via a 30-gauge needle connected to a pump, over 90s, in 1 µl, to non-anesthetized animals. We then investigated the possible involvement of Trpvs regulating the excitability of AVP neurons [39] in the basal overactivation of AVP neurons in aged rats, by treating aged rats centrally with ruthenium red (RR), a non specific Trpv blocker. RR (Tocris Bioscience, Ellisville, USA) was also dissolved in aCSF and administered, as previously described [40], i.c.v., in 1 µl, over a period of 5 min, to unanesthetized animals.

### SON immunohistochemistry

Rats were terminally anesthetized with sodium pentobarbital and transcardially perfused with PBS, pH 7.4, followed by 300 ml 4% paraformaldehyde (PFA). The brains were carefully harvested and post-fixed overnight in the same 4% PFA solution at 4°C. The following day, this solution was changed to a 30% sucrose solution prepared in PBS. The brains were then frozen and maintained at −80°C until being sectioned on a Leica CM305S cryostat at a thickness of 30 μm (Leica, Rueil Malmaison, France). Free-floating sections were collected in an anti-freeze solution and stored at −20°C until use. AVP neurons were identified by incubation with a guinea pig antiserum against AVP (Peninsula Laboratories, Bubendorf, Switzerland) diluted 1/2000, followed by the biotinylated goat anti-guinea pig antibody (Vector, Burlingame, USA) at a dilution of 1/4000 and then streptavidin Alexa 594 (Invitrogen, Cergy Pontoise, France) at a dilution of 1/4000. Astrocytes were identified by incubation with a rabbit GFAP antibody (Dako Glostrup, Denmark), diluted 1/1000, which was detected by incubation with the goat anti-rabbit Alexa 594 antibody (Invitrogen, Cergy Pontoise, France) at a dilution of 1/2000. Immature astrocytes were identified by incubation with a mouse vimentin antibody (Sigma Aldrich, Saint-Louis, USA) at a dilution of 1/1000, followed by the biotinylated horse anti-mouse antibody (Vector, Burlingame, USA) at a dilution of 1/2000 and then streptavidin Alexa 594 (Invitrogen, Cergy Pontoise, France) at a dilution of 1/2000. Activated microglia were identified by incubation with a mouse CD11b antibody (AbD Serotec, Colmar, France) at a dilution of 1/200, followed by a biotinylated horse anti-mouse antibody (Vector, Burlingame, USA) at a dilution of 1/500 and then with avidin-biotin peroxidase complex (Vector, Burlingame, USA) at a dilution of 1/1000. We then used diaminobenzidine (DAB) as a substrate to detect peroxidase activity, according to the nickel-enhanced glucose oxidase method (Shu et al 1988), which yields a black precipitate. The nonspecific cationic channels, type 2 Trpvs (Trpv2) were identified by incubation with a rabbit Trpv2 antibody (Calbiochem Merck, Darmstadt, Germany) at a dilution of 1/200, followed by the biotinylated donkey anti-rabbit antibody (Jackson, Bar Harbor, USA) at a dilution of 1/400, and then streptavidin Alexa 594 (Invitrogen, Cergy Pontoise, France) at a dilution of 1/400.

### Microscopy

Fluorescent brain sections were examined under a confocal microscope (TCS SP2; Leica, Wetzlar, Germany) or an epifluorescence microscope (DMR; Leica, Wetzlar, Germany), with appropriate filters. DAB-treated brain sections were examined under a light microscope (Nikon Eclipse E400, Nikon Corporation, Champigny-sur-Marne, France).

Determination of the nucleus volume of AVP-stained neurons: We determined the volume of the nucleus in AVP-stained neurons as previously described [41], with Image J (NCBI, Betesda, USA). Neurons are ellipsoid in shape. We therefore had to measure two axes to obtain an appropriate estimate of the volume of the neuron. The shortest (Dmin) and largest (Dmax) diameters of the cell nucleus, crossing at an angle of 90°, were measured in immunohistochemically stained AVP neurons. Cell nucleus volumes were subsequently calculated according to the formula for a prolate spheroid, π *a b* ^2^/6, where *a*, is Dmax and *b* is Dmin. Dmin, Dmax and the corresponding cell nucleus volume were calculated for each neuron separately. Nucleus volume was determined for all neurons clearly visible on four SON sections per rat, for six rats per group.

Determination of the thickness of the SON-ventral *glia limitans* (SON-VGL): We measured SON-VGL thickness, a marker of astrocyte morphofunctional plasticity, by LAS-AF (Leica, Wetzlar, Germany) on 30 µm-thick sagittal sections. We made 10 measurements across the width of each SON section. Mean values were calculated from four SON sections per rat and six rats per group.

Determination of the number of CD11b-positive microglia: CD11b-positive cells were counted manually throughout the entire SON, on 30 µm-thick sagittal sections (six rats per group).

Determination of the intensity of Trpv2 staining: the intensity of Trpv staining was determined with Image J, by applying a fixed threshold to gray images. The intensity above the threshold was then quantified for the whole SON (six rats per group).

### Determination of plasma AVP and apelin concentrations

Rats were decapitated and whole blood (4–5 ml) was collected into chilled tubes containing 0.25 ml of 0.3 M EDTA (pH 7.4) on ice and centrifuged at 1600 *g* and 4°C for 15 min.

Plasma AVP concentration was determined as previously described (Moreau et al 2010), in a specific, non radioactive, competitive binding assay (R & D Systems Europe Ltd., UK). Apelin determinations were performed by radioimmunoassay, as previously described [5], [8], with a specific apelin antiserum. Mean IC_50_ values for inhibition of the binding of [^125^I]pE13F to the apelin antiserum were 0.28±0.08 nM. The minimal concentration of pE13F required for significant displacement of the tracer was 6 fmol. The cross-reactivity of the apelin antiserum with various N- and C-terminally truncated fragments of K17F was determined. Taking reactivity with pE13F as 100%, reactivities could be ranked as follows: K17F > pE13F  =  L36F ∼ R12F > P11F > R10F > G5F >> K16P  =  K15M, with negligible reactivity observed for AngII, AngIII, neuropeptide Y, and AVP. Serial dilutions of tissue extracts and plasma samples gave a progressive inhibition of the binding of [^125^I]pE13F to the antiserum, with inhibition curves similar to that obtained with pE13F used as a standard [5].

### Bioplex determinations of cytokine levels in the SON

Rats were decapitated and their brains were immediately frozen in liquid nitrogen. Brains were sectioned and bilateral punches of the SON were collected. Cytokines (IL-1β and TNF-α) were determined with a Biorad (Hercules, CA) cytokine multiplex kit, according to the manufacturer's instructions. The sensitivity of the multiplex kit was 50 pg.ml^−1^. Data are expressed as pg of proteins per mg of total protein.

### Western blotting for GFAP in the SON

For each rat, punches of the SON from both sides of the brain were homogenized in lysis buffer and protein concentration was determined with a BCA assay kit (Uptima, Montluçon, France). Equal amounts of protein (10 µg) were run on SDS/PAGE gels (12% acrylamide) and transferred onto PVDF membranes (Millipore, Billerico, MA, USA). Membranes were incubated overnight at 4°C with anti-GFAP (1/1000, Santa Cruz Biotechnology, Santa Cruz, CA, USA) or anti-actin (1/2500, Sigma) antibodies. The membranes were then washed and incubated with peroxidase-conjugated secondary anti-goat or anti-rabbit antibody for 1 h (dilutions of 1/10000 and 1/5000, respectively, Jackson ImmunoResearch Laboratories, Westgrove, PA, USA). Between each detection reaction, the membranes were incubated for 10 min at 70°C in stripping buffer (0.065 M Tris, 1%, SDS, 0.7% β-mercaptoethanol, pH 6.7) to remove the antibodies used in the previous reaction. Antibody binding was detected with the ECL-Plus western blotting detection system (Perkin Elmer, Forest City, CA). Chemiluminescence was captured with a Syngene detection system and quantified with Gene Tools software (Syngene).

### Determination of mRNA levels in the SON

Rats were deeply anesthetized with sodium pentobarbital and transcardially perfused with heparinized phosphate-buffered saline, pH 7.4. Both SONs were rapidly microdissected and frozen in liquid nitrogen.

For CD11b, RNA was extracted in 500 µl of Trizol, according to the manufacturer's protocol (Invitrogen, Carlsbad, CA), and RNA concentrations were determined at 260 nm on a Nanodrop spectrometer (Thermo Scientific, Waltham, MA). RNA was considered to be of good quality when the area under the curve of 28S ribosomal RNA fluorescence measured on an Agilent Bioanalyzer (Agilent Technologies, Santa Clara, CA) was greater than that under the curve for 18S ribosomal RNA. In total, 2 µg of RNA was reverse-transcribed, at 42°C, with the Powerscript reverse transcriptase (Ozyme, Saint Quentin en Yvelines, France) in the presence of 1 µl of random primers (150 ng/µl) (Invitrogen, Carlsbad, CA). We then assessed the level of mRNA encoding CD11b, by real-time polymerase chain reaction (PCR) on the complementary deoxyribonucleic acid (cDNA) obtained from the SON. Primers were designed with Primer3 and their specificity was evaluated from a melting curve and their migration in a 2% agarose gel. Primers (table 1) were validated if they gave a single peak on the melting curve and a single band on electrophoresis in agarose gels. Real-time PCR was based on the quantification of SYBR green binding to double-stranded DNA in an Opticon 2 thermocycler (Bio-Rad, Hercules, CA). Real-time PCR was performed in a total volume of 10 µl, with 10 ng of the first-strand cDNA mixture as the template, 3 µl of primers and 5 µl of the reaction mixture, containing modified *Thermus brockianus* DNA polymerase, SYBR Green I dye, PCR buffer, 5 mM MgCl_2_ and a mixture of dNTPs, including dUTP (DyNamoTM SYBER Green qPCR kit, Finnzymes Oy, Espoo, Finland). The level of expression of target genes relative to that of the glyceraldehyde 3-phosphate dehydrogenase (GAPDH) gene was determined from real-time PCR efficiencies (*E*) and the threshold cycle (Ct) difference (Δ) of the sample versus a control (ΔCt _sample-control_). For AVP, apelin, IL-1β, TNF-α and Trpv2, total RNA was extracted on Qiagen RNeasy microcolumns, and contaminating DNA was degraded with Qiagen RNase-free DNase1, according to the manufacturer's instructions (Qiagen, Crawley, UK). The methods for RT-PCR and mRNA quantification have been described elsewhere [42]. Briefly, PCR primers were designed with Primer Express 1.0 software, using published sequences for rat (Sigma-Genosys Ltd, Poole, UK). All the primers designed and used in these experiments are summarized in table 1. For mRNA quantification, a standard curve was constructed from total RNA isolated from the SON (*n* = 3 rats per group). Total RNA was extracted from this tissue and cDNA was synthesized from 2 μg of RNA in a 50 μl reaction volume, with the reverse transcription reagents described elsewhere [42]. A dilution series (1/5, 1/25, 1/125, 1/625, 1/3125) of this cDNA product was then used for each SYBR green PCR, to generate a standard curve for analysis of the expression of the various genes. Results are expressed as the amount of mRNA relative to that for the housekeeping gene actin.

**Table 1:** Rat primer and probe sequences used in this paper.

TARGET GENE	PRIMER	5′-3′
CD11b	Forward	TGACGGCTCCGGTAGCAT
	Reverse	CCATCACAGTTGAGACAAATTCCT
GFAP	Forward	GGGCGAAGAAAACCGCAT
	Reverse	TCTGGAGGTTGGAGAAAGTCTGT
Vimentin	Forward	TTCCCTGAACCTGAGAGAAACTAAC
	Reverse	TCAACCAGAGGAAGTGACTCCA
Actin	Forward	TGAAGATCAAGATCATTGCTCCTC
	Reverse	AGCCACCAATCCACACAGAGT
TRPV2	Forward	GGTCTACCTGGTCTTCCTTTTCG
	Reverse	TGCTCAAGCTTACTAGGGCTACAG
VP	Forward	TGCTGCAGCGATGAGAGC
	Reverse	GAAAAAACCCTCTCGACACTCG
Apelin	Forward	CTGCTCTGGCTCTCCTTGAC
	Reverse	CATCTGGAGGCAGCATCAGT

### Experimental design and statistical analyses

Completely randomized designs were used for all experiments. Data were analyzed by t-test or ANOVA, and *post hoc* comparisons of individual group means were carried out by Fischer's LSD test for Two-way ANOVAs and Tukey's test for One-way ANOVA. In all cases, *p*<0.05 was considered to be statistically significant. All results are summarized and presented as means ± Standard Error of the Mean (SEM). We used four to six animals per group for each experiment.

## Results

### Aging prevented the dehydration-induced dual AVP/apelin response

We previously showed that AVP neurons were hyperactive in aged animals [15]. This activation occurred in the absence of a significant change in plasma osmolality, because the mean plasma osmolality values obtained for aged rats (303.4±8.8 mosmol/l, *n* = 16) were similar to those previously measured for adult rats in our experimental conditions (302, 2±2.3 mosmol/l, *n* = 35).

**Figure 1:**
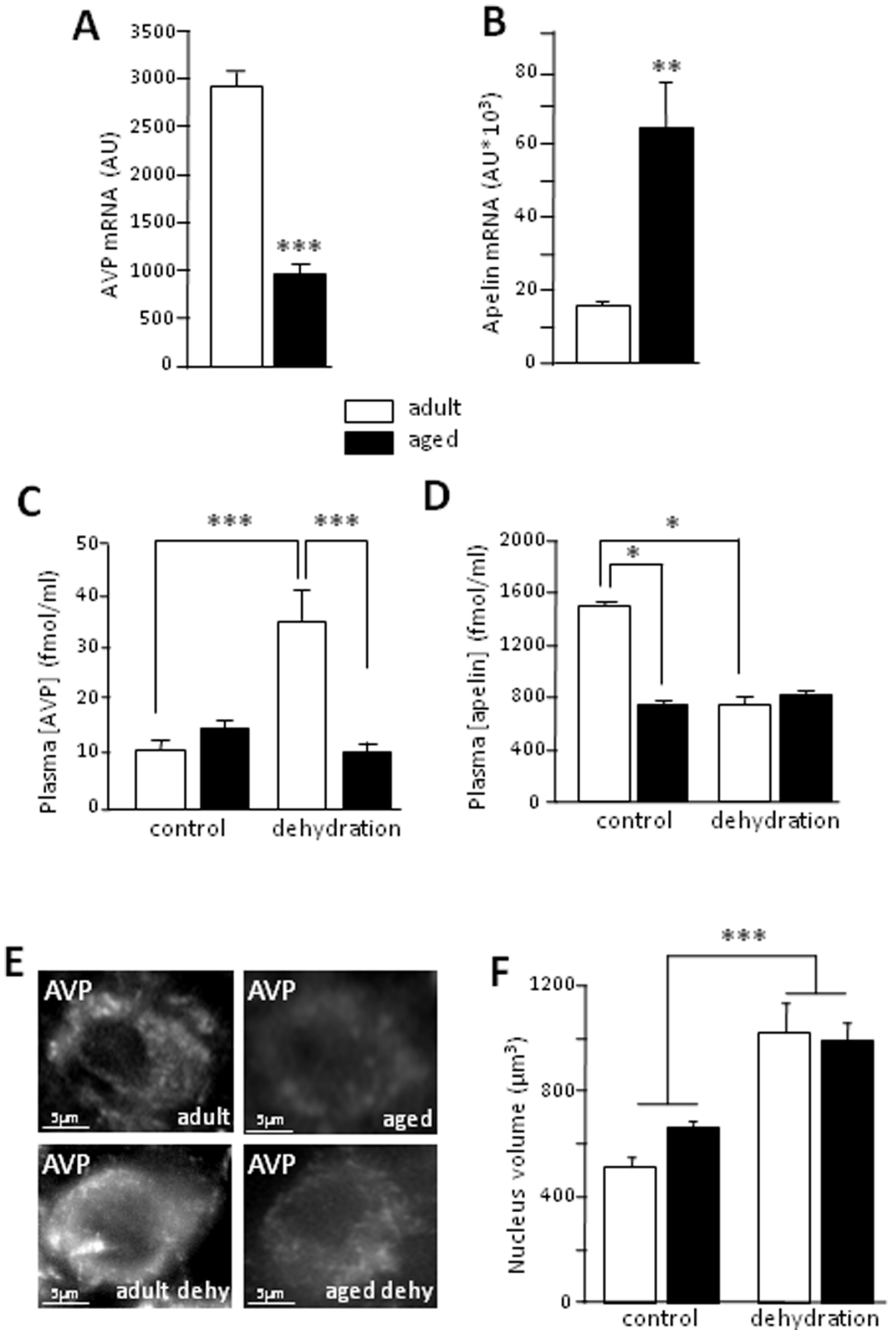
Morphofunctional characteristics of AVP neurons: influence of aging and dehydration. A- and B- Concentrations of AVP and apelin mRNA in the adult and aged SON. C- Plasma AVP concentration (in fmol/ml) in adult and aged rats under control conditions and after 48 h of dehydration (age effect, *p* = 0.042; dehydration effect, *p* = 0.034, interaction, *p* = 0.004). D- Plasma apelin concentration (in fmol/ml) in adult and aged rats under control conditions and after 48 h of dehydration. E- AVP immunohistochemistry in adult and aged rats under control conditions and after 48 h of dehydration (dehy). F-Nucleus volume of AVP neurons in adult and aged rats under control conditions or dehydration. * p<0.05; ** *p*<0.01 and *** *p*<0.001.

In the SON, AVP mRNA levels in aged rats were one third those in adult rats (Fig. 1A; *t*-test, *p*<0.001), whereas apelin mRNA levels were much higher than those in adult rats (Fig. 1B; *t*-test, *p*<0.01). We previously showed that plasma AVP concentrations were significantly higher in aged rats than in adults [11]. We now demonstrate that dehydration does not affect AVP concentrations in the same way in adult and in aged rats (Fig. 1C; Two-way ANOVA, age effect, *p* = 0.042; dehydration effect, *p* = 0.034, interaction, *p* = 0.004; Fisher's LSD *post-hoc* test, control adults vs. dehydrated adults, p<0.001, dehydrated aged rats vs. dehydrated adults, p = 0.001). Interestingly, dehydration significantly increased plasma AVP concentration in adults, but had no significant effect on plasma AVP concentration in aged rats (Fisher's LSD *post-hoc* test, *p* = 0.469) (Fig. 1C). Moreover, plasma apelin concentrations in aged rats were only half those in adults (Fig. 1D; Two-way ANOVA, age effect, *p*<0.001; dehydration effect, *p*<0.001; interaction, *p*<0.001; Fisher's LSD *post-hoc* test, control adults vs. dehydrated adults, *p*<0.001; age effect in control condition, p<0.001). Under hyperosmotic challenge, such as dehydration or hypertonic saline infusion, plasma apelin concentrations have been shown to decrease in rodents and humans [5], [8]. In our experimental conditions, dehydration for 48 h led to a significant decrease in plasma apelin concentration, by a factor of two, in adults, whereas it had no significant effect on this concentration in aged rats (Fig. 1D; Fisher's LSD *post-hoc* test, dehydration effect in aged rats, *p* = 0.336). This was not correlated to the volume of the nuclei. Indeed, in aged rats, under basal conditions, the volume of the nuclei of AVP-IR neurons was not significantly larger than in adult rats (Fig. 1E and F; Two-way ANOVA, age effect, p = 0.383). Moreover, after dehydration, the volume of the nucleus in AVP neurons was increased both in adult and aged rats to the same extent (Fig. 1E and F; Two-way ANOVA, dehydration effect p<0.001; interaction, p = 0.257).

### Aging increased the expression of neuronal Trpv2

**Figure 2:**
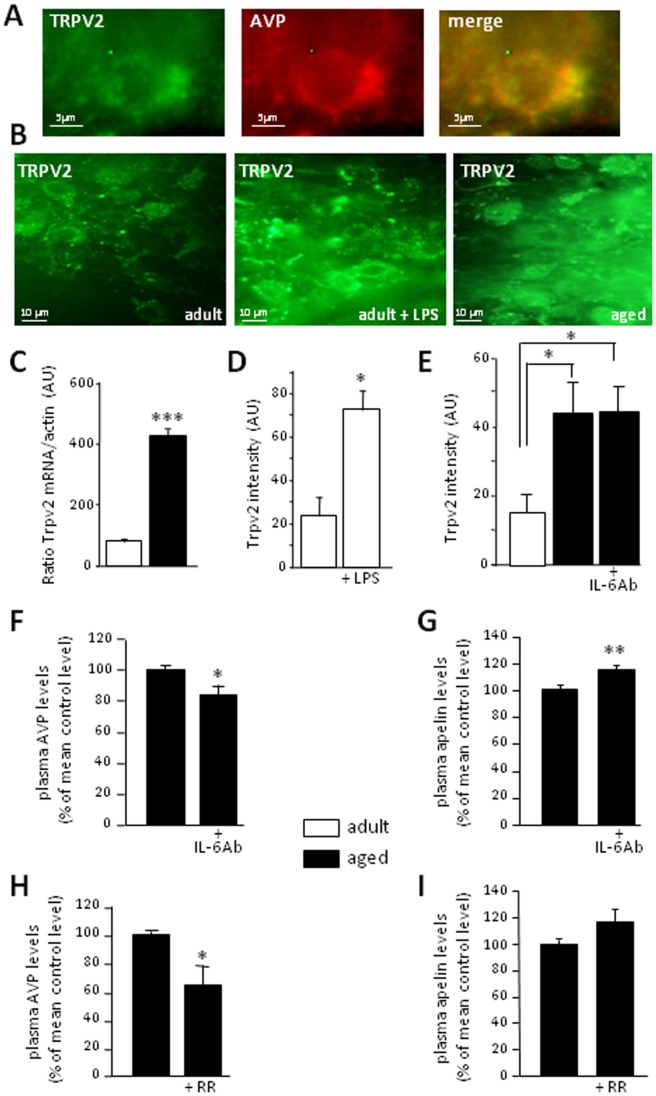
Trpv2 is responsible for AVP neuron overactivation in aged rats. A- Double immunohistochemistry of AVP neurons and Trpv2, demonstrating the expression of Trpv2 in AVP-IR neurons. B- Immunohistochemistry of Trpv2 in adult rats under control conditions or after LPS treatment, and in aged rats. C- Trpv2 mRNA levels in the SON (RT-PCR, expressed in arbitrary units, AU,) in adult and aged rats. D- Intensity of Trpv2 immunostaining (in AU) in adult rats, injected i.p. with buffer or LPS. E- Intensity of Trpv2 immunostaining (in AU) in adult rats, and in aged rats into which buffer or IL-6Ab was injected centrally. F- and G- Plasma AVP and apelin concentrations in aged rats centrally injected with buffer or IL-6Ab. H- and I-Plasma AVP and apelin concentrations in aged rats centrally injected with buffer or ruthenium red (+ RR). Plasma AVP and apelin levels in control and treated aged rats are expressed as a percentage of the mean level in control aged rats.* *p*<0.05; ** *p*<0.01; *** *p<*0.001.

We showed, by qualitative double-immunohistochemistry, that the Trpv2 channels could be found in adult rats on AVP-IR neurons (Fig. 2A) and that Trpv2 expression levels, which were low in basal conditions, were increased by LPS treatment (Fig. 2B and 2D; *p* = 0.026) and during aging (Fig. 2B and 2C; *p* = 0.05). Trpv2 mRNA levels were much higher in aged than in adult rats as well (Fig. 2C; *p*<0.001). We investigated whether Trpv2 overexpression during aging was related to the overproduction of IL-6 by astrocytes. The i.c.v. injection of IL-6 Ab had no effect on Trpv2 expression in aged rats (Fig. 2E; One-way ANOVA *p* = 0.025, Tukey's *post-hoc* test, adults vs. control aged, p = 0.041; adults vs. IL-6 Ab-treated aged, p = 0.04), but significantly decreased plasma AVP concentration (Fig. 2F; *p* = 0.038) while increasing plasma apelin concentration by about 20% (Fig. 2G; *p* = 0.009). In turn, the central treatment with ruthenium red (RR) significantly decreased AVP plasma concentration to levels similar to those classically measured in adults (Fig. 2H; *p* = 0.045). This treatment had no significant effect on plasma apelin concentration (Fig. 2I; *p* = 0.152).

### Aging prevents dehydration-induced neuroglial plasticity in the SON

**Figure 3:**
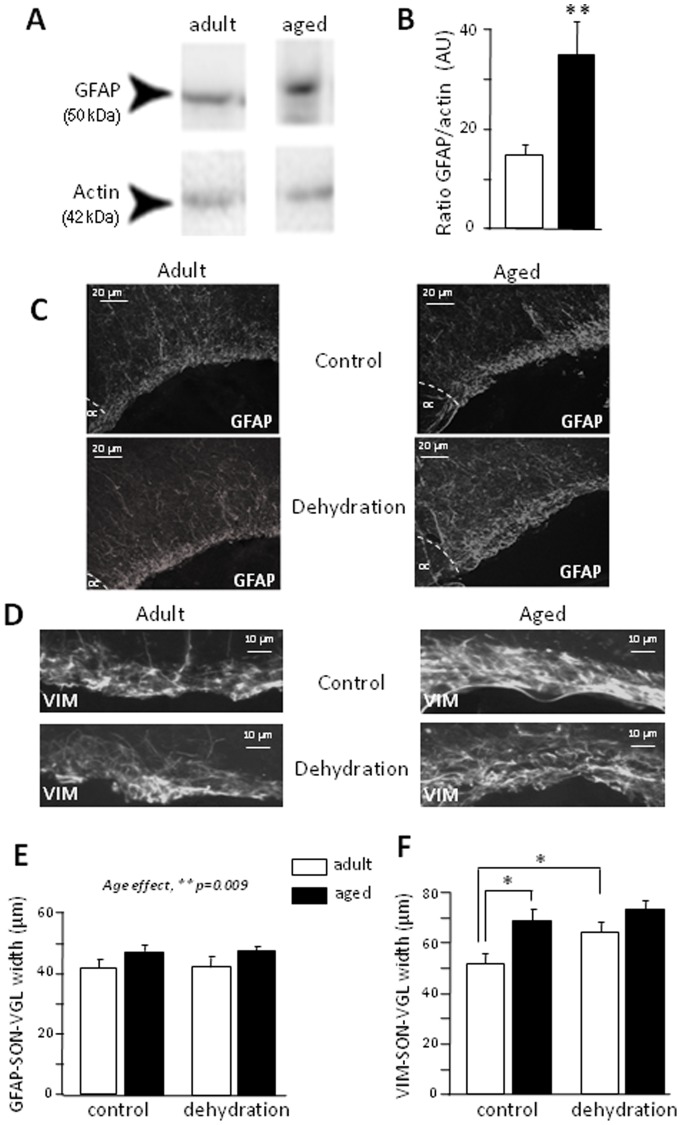
Morphofunctional characteristics of GFAP- and vimentin-positive cells during aging and dehydration: consequences for dual AVP/apelin neuron function. A- Western blot of GFAP in adult and aged rats. B-Levels of GFAP mRNA in the SON of adult and aged rats (GFAP/actin, expressed in AU). C- Immunohistochemistry of GFAP-IR cells in the SON of adult and aged rats subjected to dehydration (dehy). The dotted line indicates the limit of the optic chiasma. OC: optic chiasma. D- Immunohistochemistry of vimentin (VIM)-IR cells in the SON-VGL of adult and aged rats under control conditions and following dehydration. E- SON-VGL width, after staining for GFAP, in adult and aged rats under control conditions and following dehydration. F- SON-VGL width, after staining with vimentin (VIM), in adult and aged rats under control conditions or following dehydration. * *p*<0.05; ** *p*<0.01.

We assessed GFAP by Western blotting on the SON of aged rats under basal conditions, and compared the results obtained with those for adults. During aging, astrocytes overproduced the GFAP protein (Fig. 3A and 3B; *p* = 0.005). We then observed the morphological features of GFAP- and vimentin-IR cells in the aged SON and in that of adults normally hydrated or subjected to dehydration for 48 h (Fig. 3C and 3D). In aged rats, GFAP and vimentin labeling were mostly concentrated in the basal laminae of the SON (or Ventral Glia Limitans, VGL) (Fig. 3C and 3D). The GFAP-IR SON-VGL was significantly wider in aged than in adult rats under control conditions and after dehydration (Fig. 3E; age effect, p = 0.009; dehydration effect, p = 0.269; interaction, p = 0.594).The width of the vimentin-IR SON-VGL was significantly greater in aged than in adult rats (Fig. 3F; age effect, *p* = 0.011, dehydration effect, p = 0.038, interaction, p = 0.05; age effect in control condition, *p* = 0.003). Dehydration significantly increased the width of the vimentin-IR SON-VGL in adult rats (Fig. 3F; control adults vs. dehydrated adults, *p* = 0.004), but had no further effect in aged rats (Fig. 3F; control aged vs. dehydrated aged, *p* = 0.916).

### Aging affects the morphofunctional status of SON microglia

**Figure 4:**
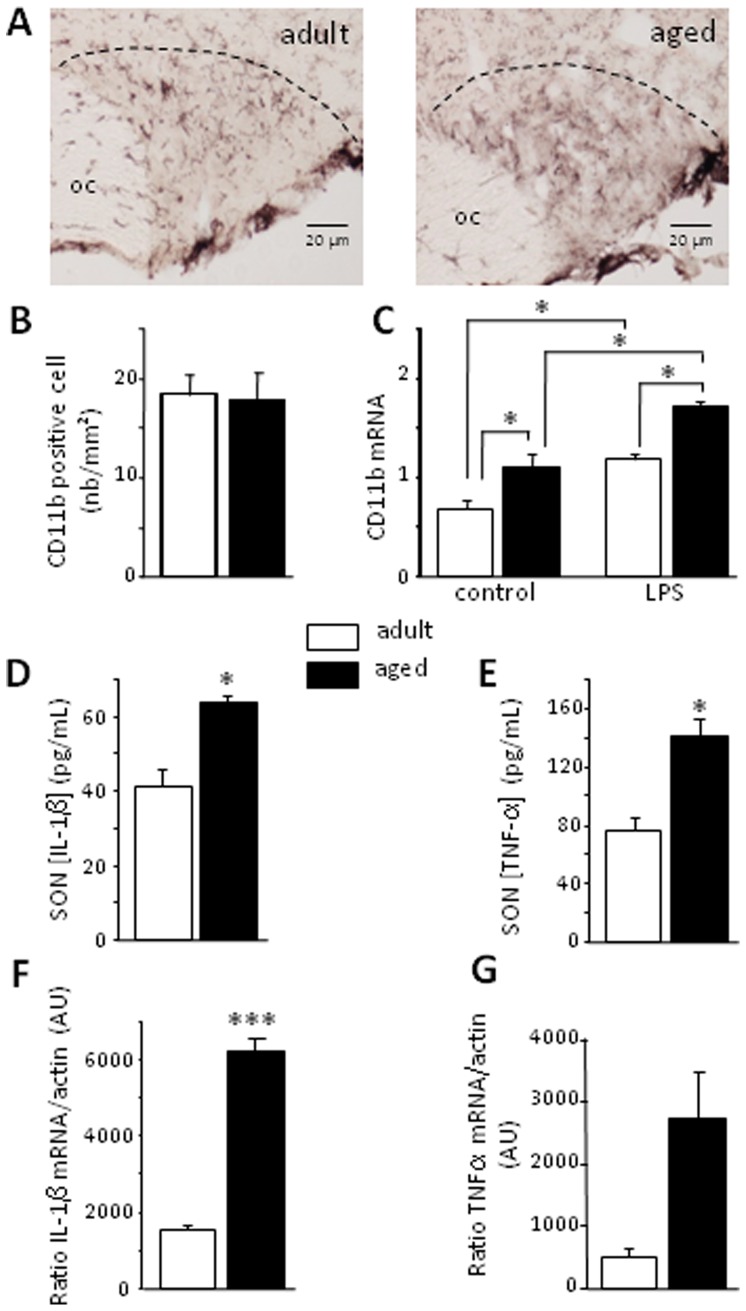
Morphofunctional state of microglial cells in aged rats. A- Immunohistochemistry of CD11b-positive cells in the SON of adult and aged rats. The dotted line indicates the limit of the SON. OC: optic chiasma. B- Number of CD11b-positive cells in adult and aged rats under control conditions. C- Levels of CD11b mRNA were determined by RT-PCR in adult and aged rats receiving i.p. injections of buffer (control) or LPS. D- and E- SON IL-1β and TNF-α concentrations, as measured by Bioplex, in adult and aged rat SON. F- and G- Levels of IL-1β and TNF-α mRNA in the SON (RT-PCR, expressed in arbitrary units, AU,) in adult and aged rats. * *p*<0.05; *** *p*<0.001.

The number of CD11b-positive cells (Fig. 4A) was similar in aged and adult rats (Fig. 4B; *p* = 0.855). CD11b mRNA levels were higher in aged rats than in adults (Fig. 4C; age effect, *p*<0.001), suggesting that microglia was activated in aged animals. Inducing a transitory inflammatory state by LPS treatment increased CD11b mRNA levels in both the adult and aged SON (Fig. 4C; treatment effect, *p*<0.001; interaction, *p* = 0.504). Finally, we assessed the levels of IL-1β and TNF-α proteins in the SON. During aging, SON IL-1β and TNF-α levels increased significantly, by about 43% with respect to those in adults (Fig. 4D; *p* = 0.033; Fig. 4E; *p* = 0.049). Moreover, SON IL-1β mRNA levels were much higher (Fig. 4F; *p*<0.001) and TNF-α mRNA levels were slightly higher (Fig. 4G; *p* = 0.059) in aged rats than in adult rats.

### During aging, an inhibition of microglial metabolism partly re-established AVP/apelin neuron activity

**Figure 5:**
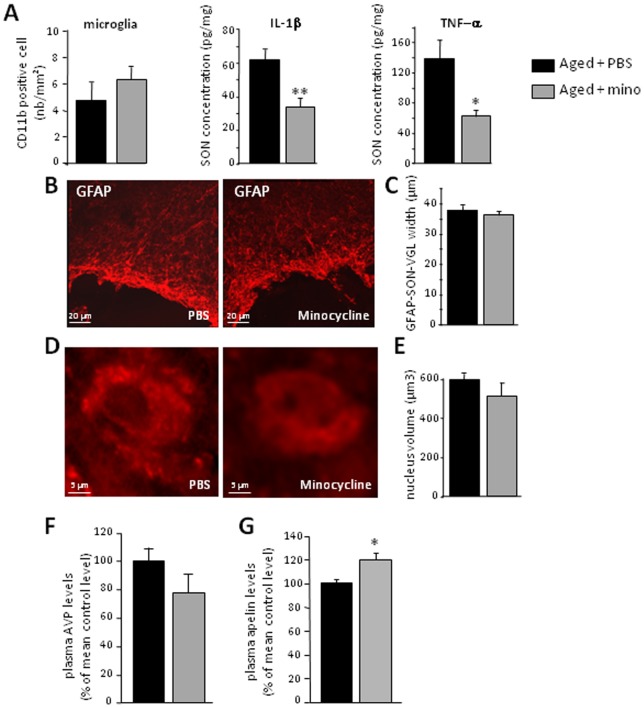
Effect of minocycline treatment on glial cells and AVP/apelin neurons in the aged SON. A- Number of CD11b-positive and concentrations (in pg/mg) of IL-1β and TNF-α in the SON, as measured by Bioplex, in aged rats previously treated with PBS (aged + PBS) or minocycline (aged + mino). B- Immunohistochemistry of GFAP-IR cells in aged rats previously treated with PBS or minocycline. C- SON-VGL width after staining for GFAP, in aged rats previously treated with PBS or minocycline. D- Immunohistochemistry of AVP-IR neurons in aged rats previously treated with PBS or minocycline. E- Volume of the nucleus in AVP-IR neurons in the SON of aged rats previously treated with PBS or minocycline. F- and G- Plasma AVP and apelin concentrations in aged rats previously treated with PBS or minocycline. Plasma AVP and apelin concentrations in control and treated aged rats are expressed as a percentage of the mean value for control aged rats. * *p*<0.05; ** *p*<0.01.

A 5-days treatment with minocycline, known to inhibit microglial metabolism, did not modify the number of CD11b-labeled cells in aged rats (Fig. 5A). More importantly, in aged rats, minocycline significantly decreased the levels of both IL-1β and TNF-α (Fig. 5A; *p* = 0.01 for IL-1beta and *p* = 0.012 for TNF-α). These findings suggest that IL-1β and TNF-α are released by activated microglia in the aged SON. By contrast, the systemic treatment of aged rats with minocycline did neither affect the width of the GFAP-IR SON-VGL (Fig. 5B and 5C; *p* = 0.597) nor the volume of the nucleus in AVP-IR neurons (Fig. 5D and 5E; *p* = 0.366). Minocycline had no effect on plasma AVP levels (Fig. 5F; *p* = 0.235), which remained similar to those in the aged control rats, but it significantly increased plasma apelin concentrations (Fig. 5G; *p* = 0.049) towards the values recorded in adults.

## Discussion

We show here that the functioning of AVP/apelin neurons changes during normal aging to maintain body fluid homeostasis, since plasma osmolality is not modified. Under basal conditions, aged neurons displayed higher plasma AVP levels and lower plasma apelin levels as in dehydrated adult rats. It is only when the aged neurons are challenged, that the impairments of the aged AVP/apelin neurons become apparent. Indeed, aged AVP/apelin neurons unable to respond suitably to a chronic hyperosmotic stimulus such dehydration. The morphofunctional plasticity of the SON neuron-astrocyte network normally observed during chronic dehydration in adults appears to be impaired in aged rats. Such impairment may partly explain the inability of elderly people to compensate for the deleterious effects of dehydration during heat waves. We investigated the mechanisms involved in age-induced neuronal adaptation and we show here, for the first time, that the sustained activation of AVP neurons is, at least in part, linked to Trpv2, the neuronal expression of which was enhanced, as in conditions of immune challenge. We also demonstrated that the overproduction of IL-6 by astrocytes and the low-grade neuroinflammation (increase in pro-inflammatory cytokine levels) induced by reactive microglial cells were likely to be involved in the dual changes in AVP/apelin neurons functioning.

### Dual change of AVP/apelin neurons during aging

In aged rats, AVP neurons displayed functional hyperactivity, as previously reported [11], [35], [43], [44], [45]. Plasma AVP levels also increased significantly during aging, as previously reported [11], [35], [43], [44], [45], [46]. We also used the total amounts of AVP and apelin mRNA as specific markers for subsequent peptide synthesis. We found that AVP mRNA levels in the SON of aged rats were lower than those in adults, whereas plasma AVP levels were higher in aged than in adult rats. By contrast, the regulation for apelin mRNA and plasma levels was opposite to that of AVP. This may reflect an homeostatic modulation of AVP/apelin neurons activity to maintain a steady-state level of neurohormons in the bloodstream.

An age-related decrease in hypothalamic and pituitary AVP levels has been reported in rodents [47], [48], suggesting that AVP turnover rates may be greater in aged rats than in adults. By contrast, apelin mRNA levels in the apelin neurons of the hypothalamus were higher in aged than in adult rats, whereas plasma apelin concentrations were lower in aged than in adult rats. We did not determine hypothalamic apelin concentrations in aged rats, but our data may also suggest a deregulation of the biosynthetic activity, storage and release of apelin in aged rats, with an opposite effect for AVP: despite low levels of AVP synthesis, AVP release into the bloodstream increases, resulting in a decrease in the AVP content of neurons, whereas apelin accumulates within neurons due to a decrease in its release and an increase in its synthesis. These opposite release profiles are similar to those previously observed in dehydrated adult rodents [5] and humans [8]. This alteration of the AVP/apelin neurons during aging may account for the inadequate response of AVP/apelin neurons to dehydration in aged animals. The high plasma AVP concentrations of aged Wistar rats were not further increased by dehydration in our conditions, by contrast to what was observed for adult rats. Moreover, the low plasma apelin concentrations observed in aged rats did not decrease further on dehydration, by contrast to what was observed for adults. This is probably because aged AVP/apelin neurons already displayed permanently high levels of AVP activation and apelin down regulation, making any further regulation of their functioning difficult. Our data are consistent with previous observations showing an attenuated response to dehydration [46], [49], [50], [51]; and immune challenge [11], and a lower sensitivity to osmotic factors [49], [50] in elderly animals. The inability of the aged rats to attain high plasma AVP concentrations during chronic water deprivation probably reflects an inability to increase AVP synthesis. This hypothesis is plausible because the AVP mRNA content of the SON does not increase with dehydration in old rats [46], [52]. Furthermore, the inability of aged rats to respond suitably to a chronic hyperosmotic challenge may be supported by apelin hypoactivity. Indeed, in adult rodents, i.c.v. apelin administration has been shown to inhibit the phasic electrical activity of AVP neurons and to reduce plasma AVP concentrations [5]. This central inhibitory feedback control, which limits the activity of AVP neurons in physiological conditions [5], [9], may be progressively impaired during aging, leading to the permanent overactivation of aged AVP neurons, resulting in a lack of control over their basal and stimulated activity.

More importantly, we demonstrated for the first time that the aging-induced overactivation of AVP neurons results from changes to their intrinsic properties, partly through the overexpression of Trpv2, a cationic channel known to regulate neuron excitability. Indeed, the central pharmacological blockage of Trpv channels by ruthenium red in aged rats leads to a significant decrease in plasma AVP concentrations, which fall toward adult values. However, it should be kept in mind that this decrease cannot be attributed exclusively to Trpv2 blockade, because ruthenium red blocks all Trpv channels. However, this effect is specific, as no effect on plasma apelin concentration was observed. LPS-induced activation of the immune system also increased Trpv2 expression in the SON, suggesting a role for inflammatory factors, as previously reported in the rat dorsal root ganglion [19]. In aged rats, IL-6 Ab treatment decreased plasma AVP concentration but had no effect on Trpv2 overexpression, suggesting that IL-6-induced AVP neuron hyperactivity is not mediated by Trpv2 expression. However, this does not rule out the possibility of an effect of IL-6 on Trpv2 activity. IL-1β and TNF-α are good candidate molecules for involvement in this effect, as they have been shown to increase Trpv1 expression in dorsal root ganglion neurons [53], [54]. Furthermore, IL-1β depolarizes magnocellular neurons *in vitro* [55], and directly excites SON magnocellular neurons via the upregulation of an osmosensory cation current [56] mediated by a member of the Trpv family. Further studies are thus required to elucidate the relationship between these pro-inflammatory cytokines and Trpv2 activity.

### Astrocyte activation during aging

During adulthood, SON astrocytes, through morphological changes affecting the extent and complexity of their processes [21], [57], have been shown to contribute to the optimal functioning of AVP/apelin neurons, which adapt their phasic pattern of discharge to ensure an appropriate response to the hormonal demand. This morphological plasticity of astrocytes is dependent on the expression of both GFAP, an intermediate filament providing cells with support and strength, and vimentin, an intermediate filament protein from immature glial cells conferring a certain degree of immaturity and morphological plasticity [24]. We therefore used immunoreactivity for both GFAP and vimentin to characterize the functionality of aged astrocytes. GFAP protein levels increase during aging, reflecting a sustained upregulation of astrocyte activity. These findings are consistent with published data for areas of the rodent brain [58], [59], [60], [61], [62], and they indicate that GFAP overexpression by astrocytes is a common feature of the aging process. In aged rats, vimentin- and GFAP-IR processes are retracted at the base of the SON, mostly in the VGL, whereas they spread throughout the SON in adult rats. The significant increase in the thickness of the vimentin-IR SON-VGL during aging may account for the failure of the neuroglial remodeling required for a dehydration-induced increase in AVP release and decrease in apelin release. The thickness of the vimentin-IR SON-VGL changed no further after 48 h of dehydration in aged rats, whereas, in adult rats, dehydration resulted in a further increase in this thickness. Overall, the data presented here suggest that, in their basal state, aged astrocytes are already overstimulated and display maximal activity. We therefore suggest that the modulation of AVP/apelin neurons activity under both basal and stimulated conditions during aging is linked to the stiffening of the overactivated astrocytes in the SON-VGL.

IL-6 was identified as a good candidate molecule expressed by aged astrocytes and able to affect AVP/apelin neuron activity. IL-6 is upregulated in the SON by dehydration in adults [63] and during aging [11]. In adult rats, brain IL-6 is known to underlie the LPS-induced early activation of AVP neurons [11] and, in aged rats, the central injection of IL-6 Ab reverses LPS-induced antidiuresis [15]. We show here that dual AVP/apelin activity during aging is dependent on IL-6, because IL-6 Ab treatment significantly decreased AVP release and increased the release of apelin. However, the recovery was only partial, suggesting that IL-6 was probably not the only factor involved. The decrease in IGF-I production by astrocytes during aging [15], [64] may also sustain AVP neuron activity, as previously demonstrated [64].

### Microglial cell dysfunction during aging

Our data clearly show that the number of CD11b-IR cells (corresponding to activated microglia) in the SON is not affected by aging. This result is not consistent with other reports of an age-related increase in the number of microglial cells [29] and in the activation of microglial cells [28], [65] with an ameboid morphology [65]. The microglial cells seemed to retain their adult morphology in the SON (3D morphology reconstitution, preliminary data not shown). By contrast, we observed an age-related increase in both the basal and LPS-induced production of CD11b mRNA in the SON, as previously described for the hippocampus [66]. The increase in CD11b mRNA levels in SON was associated with an increase in SON IL-1β and TNF-α levels, as in other brain areas [67], [68]. These changes are thought to be markers of a chronic state of low-grade inflammation [69], [70]. We tested this hypothesis in the SON, by treating aged rats with minocycline, an inhibitor of microglial metabolism, which is known to inhibit several aspects of the inflammatory process [71]. Treatment for five days induced a decrease in the concentrations of IL-1β and TNF-α in the SON. By contrast, minocycline affected neither the morphology of aged astrocytes nor SON-VGL thickness and AVP-IR neuron morphology (no change in nucleus volume). Instead, it increased plasma apelin concentration, suggesting a substantial role for microglia, possibly through IL-1β and TNF-α, in the dual AVP/apelin neuron activity. This effect may be exerted directly on neurons rather than via the astrocytes, as suggested by previous data showing that IL-1β depolarizes magnocellular neurons *in vitro* [55], directly exciting SON magnocellular neurons via the upregulation of the osmosensory cation current [56].

## Conclusion

In conclusion, there is a dual adaptation of AVP/apelin neurons during aging to maintain body fluid homeostasis, as we found that plasma vasopressin concentrations were higher and plasma apelin concentrations lower, in aged rats than in younger adults. However, the response of AVP/apelin neurons to osmotic challenge is impaired. Such dysfunction could find an origin in the sustained high plasma AVP level and low apelin level observed under basal conditions. AVP is highly released into the bloodstream, whereas apelin accumulates within AVP neurons rather than being released, as observed following water deprivation. These complex changes have their origin in the neurons themselves. Our data suggest that AVP neuron hyperactivity is partly sustained by neuronal Trpv2 overexpression, as observed during immune challenge. Thus, there may be an absence of additional up- and down regulation of AVP and apelin in response to a sustained osmotic challenge, as the neuronal system may already be functioning at maximal capacity. Glial partners are also involved in the dual AVP/apelin neuron adaptation, through the overproduction, by astrocytes, of IL-6 and the overproduction of IL-1β and TNF-α, in particular. Finally, the lack of morphofunctional plasticity in stiffened aged astrocytes at the base of the SON, resulting in defective physiological responses in adults, may also account for the inability of magnocellular AVP/apelin neurons to respond to physiological stimuli. This astrocyte dysfunction is not a secondary effect of microglial activation.

Several putative mechanisms can be proposed in the differential functions of AVP and apelin neurons. The existence of separate vesicle populations in conditions of apelin and AVP coexistence [9] implies that stimulus paradigms are used by the organism *in vivo*, allowing the selective release of only one of the two vesicle populations. It is well known that the sustained AVP release during dehydration is frequency-coding dependent [72]. The possible release mechanisms for apelin during water loading [8] may involve low frequency coding (low frequency; [73]) or constitutive regulation like for insulin [74] (see discussion in [9]). The constitutive secretion of apelin would requires an absence of extracellular calcium, which is unlikely in aged AVP neurons, even after the blocking of Trpvs with ruthenium red. We suggest that the overexpression of Trpv2 in aged rats may contribute to neuron depolarization via Ca^2+^ entry, increasing spike frequency, thereby facilitating the release of AVP but not apelin. This appears plausible, because the Trpv blockade with ruthenium red in aged rats led to a return of AVP secretion to adult levels, probably reflecting a restoration of basal electrical activity to levels still not compatible with apelin release.
